# A computational investigation of feedforward and feedback processing in metacontrast backward masking

**DOI:** 10.3389/fpsyg.2015.00006

**Published:** 2015-02-24

**Authors:** David N. Silverstein

**Affiliations:** ^1^PDC Center For High Performance Computing, KTH Royal Institute of TechnologyStockholm, Sweden; ^2^Department of Computational Biology, KTH Royal Institute of TechnologyStockholm, Sweden; ^3^Stockholm Brain Institute, Karolinska InstituteSolna, Sweden

**Keywords:** backward masking, visual cortex, feedback projections, conscious processing, neural attractor dynamics

## Abstract

In human perception studies, visual backward masking has been used to understand the temporal dynamics of subliminal vs. conscious perception. When a brief target stimulus is followed by a masking stimulus after a short interval of <100 ms, performance on the target is impaired when the target and mask are in close spatial proximity. While the psychophysical properties of backward masking have been studied extensively, there is still debate on the underlying cortical dynamics. One prevailing theory suggests that the impairment of target performance due to the mask is the result of lateral inhibition between the target and mask in feedforward processing. Another prevailing theory suggests that this impairment is due to the interruption of feedback processing of the target by the mask. This computational study demonstrates that both aspects of these theories may be correct. Using a biophysical model of V1 and V2, visual processing was modeled as interacting neocortical attractors, which must propagate up the visual stream. If an activating target attractor in V1 is quiesced enough with lateral inhibition from a mask, or not reinforced by recurrent feedback, it is more likely to burn out before becoming fully active and progressing through V2 and beyond. Results are presented which simulate metacontrast backward masking with an increasing stimulus interval and with the presence and absence of feedback activity. This showed that recurrent feedback diminishes backward masking effects and can make conscious perception more likely. One model configuration presented a metacontrast noise mask in the same hypercolumns as the target, and produced type-A masking. A second model configuration presented a target line with two parallel adjacent masking lines, and produced type-B masking. Future work should examine how the model extends to more complex spatial mask configurations.

## Introduction

Visual backward masking is a classic technique used to examine differences between conscious and unconscious visual processing (Breitmeyer and Ogmen, 2006). It is employed by presenting a target image followed closely in time by a mask image. The target image exposure is typically very short, often around 20 or 16.7 ms, but may be limited by monitor refresh rates. The mask typically has longer exposure, often at least 50 ms, but sometimes up to hundreds of milliseconds. The time from the start of the target exposure to the time of the start of the mask is experimentally varied, and this is commonly known as the stimulus onset asynchrony (SOA). While there are many experimental variations, target and mask exposure times often remain fixed while the SOA is varied. When the SOA is 20–60 ms, a face target is sometimes not consciously perceived (Rolls, 2004). The measured response from recognizing a masked target has been characterized as type-A and type-B masking. In type-A masking, the masking effect monotonically decreases with increasing SOA. This is often associated with a stronger masking stimulus. In type-B masking, the masking effect is weaker at low SOAs, becomes stronger at some point with SOAs less than 100 ms and then diminishes again with increasing SOA, with a response curve sometimes referred to as a U-shaped function (Breitmeyer and Ganz, 1976). Different types of masks are possible. Pattern masking occurs when the mask shares some features with the target or is superimposed. Metacontrast masking occurs when the mask features are non-overlapping with the target, but some features may be in close spatial proximity. Masks may also be different forms of noise, and might also be a flash of light (Breitmeyer and Ogmen, 2000).

There are two broad classes of conceptual models for explaining backward masking. One states that visual sensory information is stored in a visual sensory buffer (or iconic memory) for processing, but can be interrupted by a mask (Sperling, 1963; Di Lollo, 1980). The other states that information propagates in dual channels (such as parvocellular and magnocellular pathways), with one faster and more transient and the other slower and more sustained. When the target and mask are presented to both channels, the fast transient activity of the mask suppresses the slow sustained activity of the target through inter-channel inhibition. The psychophysics of masking have been characterized, although individual differences have been observed in stable masking functions (Albrecht and Mattler, 2012). Less understood are the underlying cortical dynamics, which are still deeper in debate (Macknick and Martinez-Conde, 2007). There are several prevailing theories on the mechanisms of backward masking and visual masking in general. One view states that this is primarily caused by feedforward lateral inhibition (Macknick, 2006). The mask spatiotemporally interferes with the target through inhibition, preventing further processing. Another view asserts that the mask interferes with feedback processing from higher areas, preventing the discrimination between the figure and background which makes visual awareness possible (Lamme and Roelfsema, 2000; Super et al., 2001; Lamme et al., 2002).

Several computational models have been developed over time and at different levels of abstraction, a subset of which will be discussed here. Earlier models focused more on the temporal aspects of the masking function, with later models incorporating some spatial aspects as well (Francis, 1997, 2009; Hermens et al., 2008). The retino-cortical dynamics (RECOD) model (Ogmen, 1993) is a dual-channel approach which incorporates neural representations as well as feedforward dynamics and feedback inhibition. It utilizes transient-on-sustained inhibition to explain some backward masking properties (Breitmeyer and Ogmen, 2000). The Boundary Contour System (BCS) originally developed by Grossberg and Mingolla (1985) and extended by Francis (1997) can reproduce many aspects of metacontrast masking. It uses model neurons, can spatially represent two orientation preferences and includes elements of lateral inhibition and feedback. Bugmann and Taylor (2005) also developed a detailed neural model with feedforward and lateral connections, which was able to produce U-shaped masking functions. Spatial aspects of backward masking have also been explored by modeling the shine-through effect (Herzog et al., 2001). When a vernier target with two adjacent and offset vertical lines is masked by a grating with five straight lines, target perception is impaired. However, if masked with a grating of seven or more straight lines, the vernier target is more easily perceived, and “shines through” the grating. The 3D-LAMINART (Grossberg, 1997; Francis, 2009) and WCTM (Herzog et al., 2003) computational models have been able to reproduce some but not all aspects of these phenomena (Rüter et al., 2011). 3D-LAMINART is a general purpose visual model that utilizes binocular vision to perceive the vernier target. WCTM is a simpler two-layer model which uses lateral inhibition to suppress repeating patterns such as lines.

This study seeks to model the cortical dynamics of metacontrast backward masking at a biophysically detailed level, to investigate the roles of feedforward, feedback and lateral connections, specifically in the context of interacting neural attractor networks (Hopfield, 1982; Amit, 1989; Hertz et al., 1991). This spiking neural attractor model is conceptually related to the sensory store model or iconic memory, because a neural attractor is a recurrent store of activity for associative processing. Among existing neural models (Francis, 1997, 2009; Bugmann and Taylor, 2005) the work presented here is perhaps the most biophysically detailed cortical model to date used to simulate the temporal aspects of backward masking. The spatial aspects are currently limited to abstract metacontrast representations where the target and mask are represented in close proximity in common hypercolumns or as parallel lines, although this could be extended with biophysical feature detectors (Rehn et al., 2011). A neural attractor in this case is considered an activated stored memory pattern, which is a neural assembly of sparse and distributed pyramidal cells recurrently connected with excitatory synapses. When a stored memory pattern (or attractor memory) is partially stimulated, it can become fully active across the distributed representation through recurrent excitation. Over time, it adapts and burns out, due to short-term synaptic plasticity and calcium dynamics, both of which can have near-second time constants. Many attractor memories can co-exist in the same neural population, and may mutually exclude each other when activated, through lateral and di-synaptic inhibition. These neural attractor memories can also activate each other associatively when overlapping and be nested and hierarchical as well. In the case of primary visual cortex, these attractor memories can represent features as grouped orientation preferences. It is hypothesized that targets consist of a set of feature detectors in individual visual areas, each with an associated patch-level attractor memory, containing minicolumns that are themselves small-world networks and mini-attractors. These patch-level (i.e., V1 or V2) attractor memories are interconnected across visual areas, activating regional-level attractors through feedforward and feedback projections. With feedforward activity, attractor activations propagate up the ventral stream (Kravits et al., 2013) as a traveling wave (Sato et al., 2012), while feedback activity provides competitive reinforcement from previous perceptual memories, or resolves ambiguity and expectation partially on the regional level (Wyatte et al., 2014). Eventually, this traveling wave is postulated to reach the pre-frontal cortex for global-level attractor activation or “ignition” for conscious access (Dehaene and Changeux, 2011). The model in this study hypothesizes that regional-level attractor memories exist across V1 and V2 and is limited to those areas. When a patch-level attractor memory is stimulated, it takes time for recurrence to fully activate it, sometimes up to 50 ms. During this time, it can be more vulnerable to interference such as a metacontrast noise mask, which may produce a monotonically decreasing masking function as the attractor builds and becomes more stable. If two competing attractor memories are activated as a target and mask, the interference between them can build as the attractors build, depending on spatial overlap or proximal contours. With spatial overlap, it is hypothesized that activation is more likely to transition to the masking attractor memory, if the target is not reinforced by feedback. In common-offset masking where the target and mask are presented simultaneously, transitions to the masking attractor memory can also occur (Enns and Di Lollo, 2000). Proximal contours during masking may also interfere with target attractor activation via lateral inhibition.

Evidence suggests that the latency of projections between V1 and V2 is about 10 ms in both directions (Nowak and Bullier, 1997; Girard et al., 2001), while horizontal propagation has been found to be significantly slower (Sugihara et al., 2011). This suggests that, considering the synaptic integration delays in V2, feedback to V1 may arrive before lateral processing is complete. Thus, this feedback may also be a factor in how that lateral processing completes. Both excitation and inhibition have been identified in both feedforward and feedback projections in rat primary visual cortex, although feedback inhibition appears to be less (Shao and Burkhalter, 1996). If feedback recurrently excites currently activated features, the target feature attractors will be enhanced, and be more likely to become fully active and propagate. However, if excitatory feedback were to activate attractor memories for features not present in the target, the target attractor could be inhibited through competition. Alternatively, if feedback inhibits other feature attractors through di-synaptic inhibition, then the target attractor will be enhanced through lower competition or suppressed noise, or at least would not be diminished.

## Materials and methods

**Figure 1:**
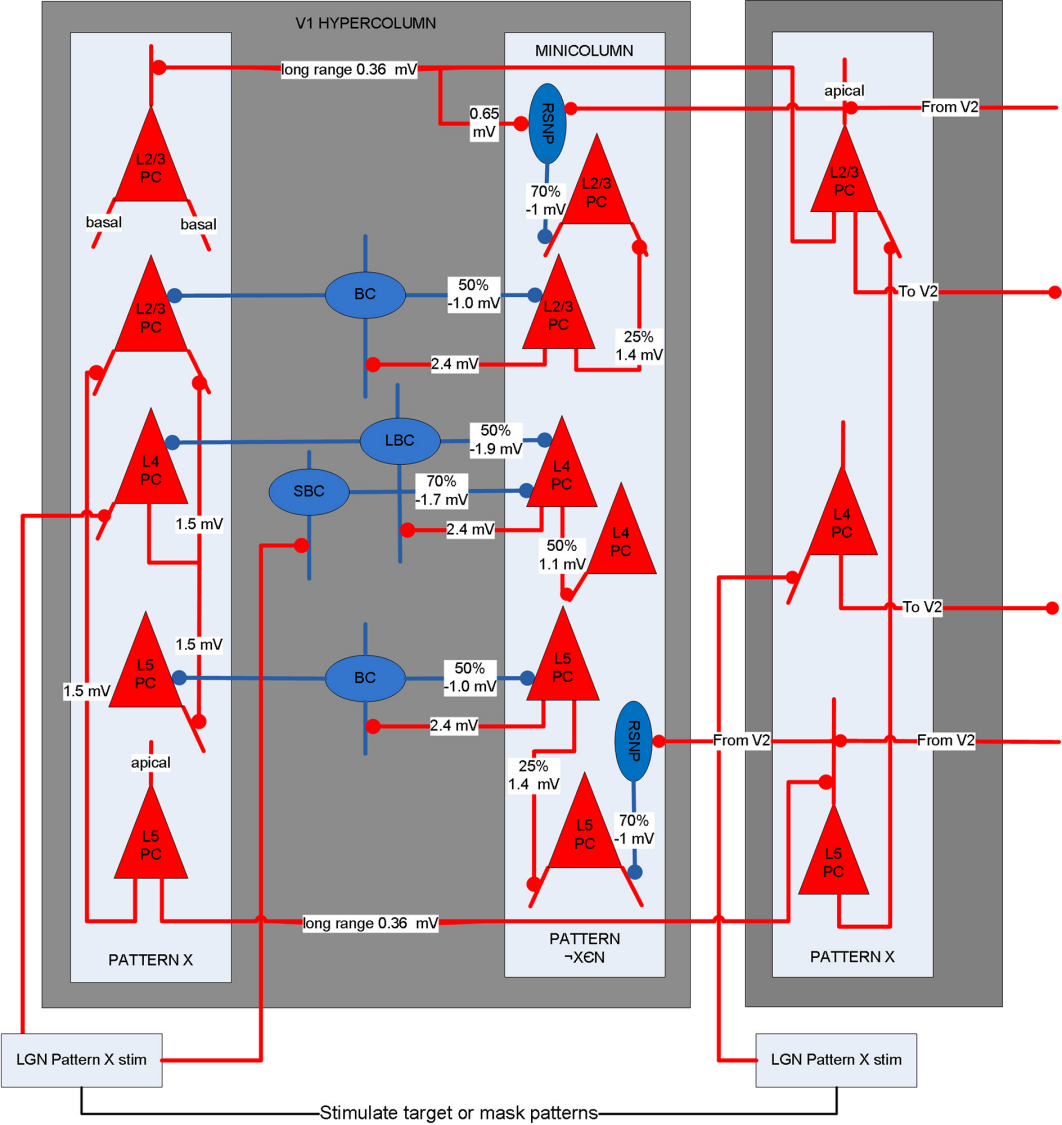
**Microcircuit of layer 2/3, 4, and 5 of V1**. Shows two minicolumns part of an arbitrary attractor memory pattern X (one of N total) in two different hypercolumns and a minicolumn outside of pattern X. Lateral inhibition from basket cells occurs within the hypercolumn between pattern X and other minicolumns. Long-range connections exist between pyramidal cells in minicolumns of the same memory pattern. Long-range di-synaptic inhibition can occur via RSNP interneurons when attractor memories have common hypercolumns. A percentage refers to the probability that a pre-synaptic population is connected to a post-synaptic population.

**Figure 2:**
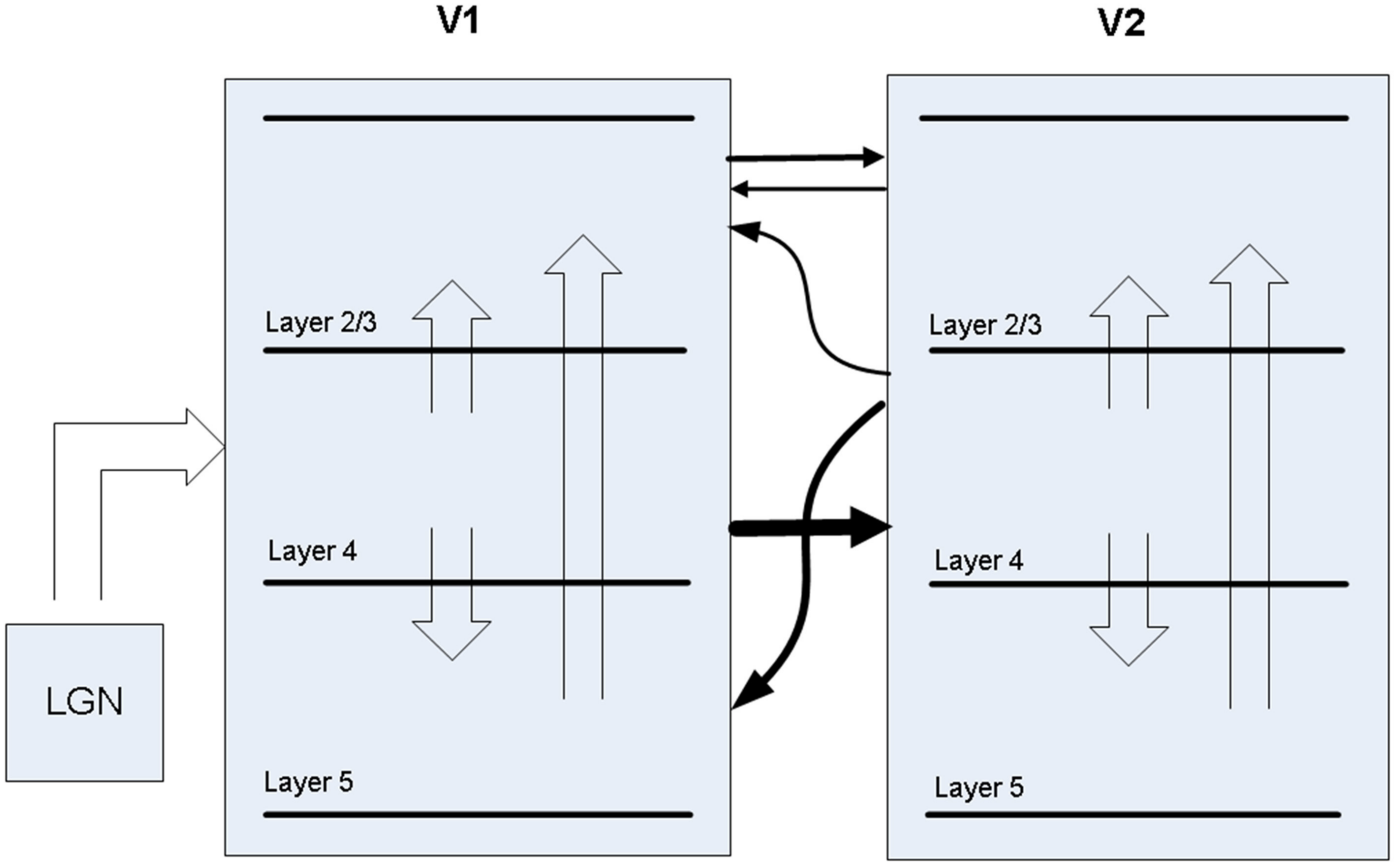
**Projections between LGN, V1, and V2 in the model**.

A biophysical model was constructed of early visual cortex, with two different instantiations. The first instantiation (called model 1) entailed using an abstract target and metacontrast noise mask in close spatial proximity. The second instantiation (called model 2) entailed using a single vertical line for the target and two adjacent parallel lines for the mask, with the intention of a more specific spatial representation. The models represent a subset of the ventral stream of primate visual cortex and includes the lateral geniculate nucleus (LGN), areas V1 and V2 and the projections between them. While projections between the LGN and V1 layer 4 are feedforward only, V1 and V2 are bidirectionally connected. The LGN is represented as a grid of 256 locations in model 1 and 648 locations in model 2, each containing a stack of 10 relay cells, acting as on-center cells. Stimuli presented to the LGN are not actual images, but are abstract representations. Off-center cells were not included. Each LGN location projects to pyramidal cells in one minicolumn of V1 layer 4 and surrounding interneurons (small basket cells), which in turn inhibit pyramidal cells in surrounding minicolumns within the same hypercolumn. The neocortical patches of V1 and V2 represent a square matrix of hypercolumns, each containing internal minicolumns. In model 1, the 4 mm^2^ patch of cortex is composed of 4 × 4 hypercolumns, subsampled with 16 minicolumns each. In model 2, the 20 mm^2^ patch of cortex is composed of 9 × 9 hypercolumns, subsampled with eight minicolumns each. The structure is similar to Silverstein and Lansner (2011), with the addition of a regular spiking non-pyramidal (RSNP) interneuron into the neocortical microcircuit (Lundqvist et al., 2006), a more complete layer 4 and the addition of layer 5. Di-synaptic inhibition and competition from RSNP interneurons occurred when attractor memories had intersecting hypercolumns, which occurred in model 1 but not model 2. The microcircuit of V1 is illustrated in Figure 1. The minicolumns are also subsampled, and contain pyramidal cells and interneurons for layers 2/3, 4, and 5. Each layer contains 20 pyramidal cells, two basket cells, and two interneurons allocated per minicolumn, although the basket cells physically reside outside the minicolumn. V1 layer 4 is known to largely contain spiny stellate cells, but pyramidal cells are used in their place for simplicity. While V1 and V2 are known to have different structure, they are both thought to have hypercolumns (Ts'O et al., 2009) and the same structure was used for both here. The V1 model represents interblobs (or interpatches) for orientation as hypercolumns, but does not include blobs (or patches) for color. It is also monocular, so does not include binocular stripes. Orientation preferences are represented in minicolumns. In model 1, these orientations remain abstract and are not tuned to particular feature preferences. However, randomly selected minicolumns in different hypercolumns are connected in stored memory patterns, representing linked orientation preferences for feature detection. While abstract, it is meant to generally represent features. In model 2, minicolumns have vertical orientation preferences for the more specific representation of line detection. V2 is known to have thin, pale and thick stripes, and the model represents the pale stripes only, which are known to also project to V4 and on along the ventral stream. Feed-forward projection streams from V1 interblobs to V2 pale stripes have been identified in Macaque (Sincich and Horton, 2005; Federer et al., 2013). These include projections from layer 2/3 and 4 of V1 interblobs to layer 2/3 and 4 of V2 pale stripes (Federer et al., 2013). Feedback projections from V2 originate from layer 2/3 and 6 and target layers 1, 2/3, and 5 of V1 (Sincich and Horton, 2005). A subset of these projections have been implemented, as can be seen in Figure 2.

Between V1 and V2, the model has feed-forward projections from V1 layer 4 to V2 layer 4 in addition to weaker projections from V1 layer 2/3 to V2 layer 2/3. Feed-back projections from V2 are predominantly from layer 4 to V1 layer 5, but also include layer 4 to V1 layer 2/3, which are about 10% of the strength. While anatomical data suggests most V2 feedback originates in layer 3, layer 4 is used for simplicity, considering dendrites from layer 3 pyramidal cells are likely to drop down into layer 4, where early activations are likely to occur after target presentation. The latencies of all projections between V1 and V2 projections are set to 10 ms, based on the findings mentioned earlier.

The model contained four different types of cells, which included spiking pyramidal cells, basket cells, RSNP interneurons and relay cells, all of which utilized the Hodgkin-Huxley formalism. The equations and parameters for these neurons are included in the Appendix. The pyramidal cells contained compartments for the soma, initial segment, basal dendrite, and apical dendrite, while the rest contained compartments for the soma, initial segment, and dendrite. With calcium dynamics, the pyramidal cells were adapting, the RSNP interneurons were weakly adapting, and the rest were not. The pyramidal cells and RSNP interneurons had Kainate/AMPA, NMDA, and GABA_A_ channels, while the basket cells had Kainate/AMPA and GABA_A_ channels. All synaptic channels had synaptic depression. However, the relay cells were stimulated only through a time-activated noise source applied to an alpha channel on the soma, and only projected to Kainate/AMPA and NMDA channels on V1 layer 4 pyramidal cells. All but the relay cells received 300 Hz of background Poisson noise and produced a positive bias.

**Figure 3:**
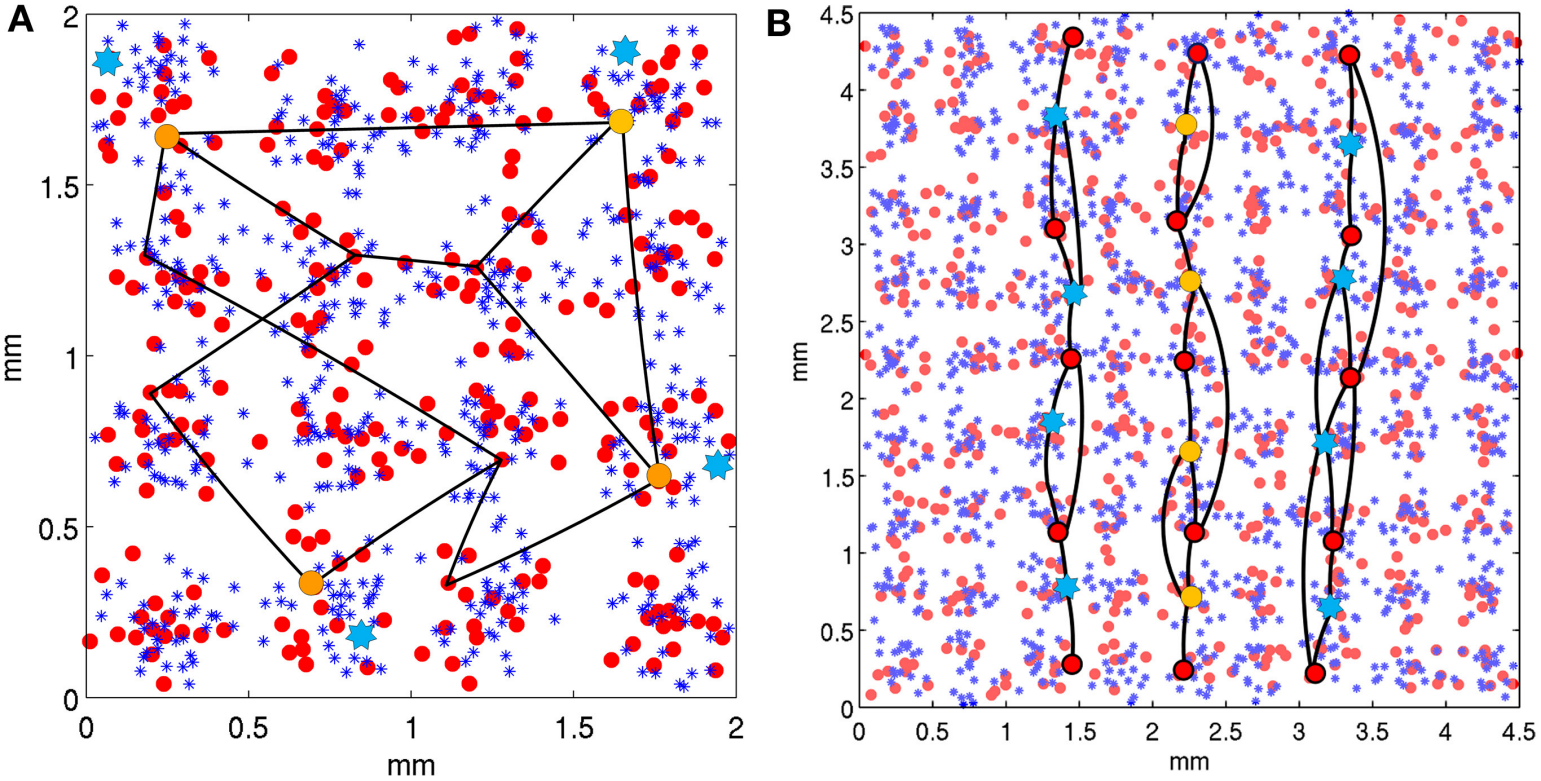
**Neocortical patches of V1 for two model configurations**. Within hypercolumns (1/2 mm in diameter) are minicolumns shown as small red circles and basket cells shown as blue asterisks. Example stored attractor memories are illustrated as black lines, which connect single minicolumns (via internal pyramidal cells) in independent hypercolumns, with a uniform connection probability. Only several of many connections of these attractor memories are illustrated. In a backward masking trial, minicolumns at orange circles are stimulated as the target and blue stars are stimulated as the mask, both via the LGN. **(A)** Shows the model 1 configuration with 16 hypercolumns, each containing 16 minicolumns. Stored attractor memories are 10 random minicolumns in separate hypercolumns across the patch. Mask stimulation occurs in the same hypercolumns as target stimulation. **(B)** Shows the model 2 configuration with 81 hypercolumns, each containing 8 minicolumns. The stored attractor memories contain 9 minicolumns each and are organized as vertical lines across hypercolumns. The target is activated as the middle vertical line and the mask is activated as the two adjacent parallel lines two hypercolumns away.

In model 1, each area had a total of 18 stored attractor memories. Each attractor memory was created by randomly choosing one minicolumn from 10 of the 16 hypercolumns, an example of which can be seen in Figure 3A. The minicolumn sampling was restricted to prevent a minicolumn from being chosen in more than one memory pattern, making the memories sparse and orthogonal. In model 2, each area had a total of 72 stored attractor memories, each containing nine minicolumns across the 81 hypercolumns, and organized as vertical lines. Once the minicolumns in an attractor memory were selected, long-range connections were created between them within the patch, which included both excitatory and inhibitory synapses. If a pairwise connection probability determined that two minicolumns in a stored memory pattern are to be connected, a pyramidal cell in the source minicolumn was randomly chosen to originate the axon. In the destination minicolumn, pyramidals received synapses with a 25% probability, and di-synaptic interneurons received synapses on surrounding minicolumns. All excitatory synapses had the same conductance, as did all the inhibitory (di-synaptic) synapses. For projections, attractor memories were connected across areas, similar to the descriptions in Szalisznyo et al. (2013). To connect two attractor memories in two different areas, the minicolumns of the memory pattern in the source area projected axons to the minicolumns of the memory pattern in the destination area. These pattern projections were not all-to-all since it was assumed that projections are only a cue to activate a remote attractor memory that would necessarily have further local representations. Thus, four minicolumns in the corresponding attractor memory were selected on the destination side to receive the axons of the pattern projection.

### Backward masking simulation

To present a target or mask stimulus to the model, 4 LGN locations, each with 10 relay cells in the LGN patch were stimulated, activating 40 relay cells in total. The target stimulus appears to the model as four dots in the grid and is sparse, representing 40% of the full target. The length of the target stimulation was always 20 ms and the length of the mask stimulation was 60 ms in model 1 and 50 ms in model 2. It was assumed that LGN relay cells fire at about 50 Hz, which meant each relay cell in a presented target stimulation would fire once over 20 ms and each relay cell included in the mask stimulation would fire three times over a 60 ms stimulation. These cell firings were uniformly distributed over the stimulation intervals. The relay cells in turn project to and stimulate minicolumns in V1, as can be seen in Figure 3. In the case of a target, the minicolumns are part of a stored memory pattern representing a feature detector, distributed across hypercolumns. In the case of a metacontrast noise mask, they are minicolumns selected from different attractor memories other than the target, which corresponds to parts of uncorrelated features. In the case of competing metacontrast line masks, the selected minicolumns were from a single attractor memories as the target was.

In model 1 as seen in Figure 3A, both the target and mask were presented as stimulated minicolumns in common hypercolumns for spatial proximity, which would roughly correspond to a visual angle of within about 10 min. Simulations were performed on model 2 with modifications for additional spatial context, to use lines in one dimension for both the target and mask, similar to stimuli presented in Growney et al. (1977). As seen in Figure 3B, the target was presented as a single, straight broken vertical line, and the mask was presented as two broken vertical parallel lines, flanking both sizes of the target and equidistant from it. The patch size was changed from model 1 to 9 × 9 hypercolumns to accommodate the short lines, with eight minicolumns per hypercolumn. The feature detectors, as attractor memories, where modified (from random assembly) to assemble selected minicolumns (as orientation preferences) vertically, along each column of hypercolumns in the 9 × 9 matrix. Each of the eight minicolumns in every hypercolumn was used in a single independent, vertically oriented feature detector, creating a total of 72 attractor memory patterns. These feature detectors were spatially redundant, but implemented so that an individual corresponding target or mask feature detector was activated for only one SOA interval during a trial run, which consisted of multiple sliding SOA intervals. This was done because the attractor memories did not completely recover from adaptation between the selective SOAs tested during each cortical second of each trial run, so couldn't be reused during a following SOA interval. Lateral inhibition in model 1 was within the hypercolumn only, but was changed to extend beyond the hypercolumn horizontally in model 2, for competition between the vertical target and mask lines. Lateral inhibition beyond the hypercolumn had a reduced basket-pyramidal synaptic connection probability of 50% one hypercolumn away, 25% two hypercolumns away and 0% outside of this. Simulations were performed with the mask 1, 2, and 3 hypercolumns away, which roughly corresponds to a fovea visual field arc of 10–20, 20–30, and 30–40 min. respectively.

For each model, five different individuals were simulated by generating 5 different neural sets, connection matrices and projections for the LGN, V1, and V2. Each of these individual instantiations was simulated for five trials with different seeds, for a total of 25 trials per trial set. Each trial consisted of presenting the target alone, the mask alone, and both target and mask with a sliding SOA of 20, 40, 60, 80, 100, and 120 ms. Feature attractors can become fully activated in Layer 2/3 and/or 5 of V1 and/or V2.

It is assumed that for the possibility of conscious perception, the linked attractor memory patterns must become fully active in layer 2/3 of both V1 and V2, indicating regional activation. To determine if this occurred, layer 2/3 of V1 and V2 were analyzed on each trial. For the attractor pattern to be considered fully activated or complete in each area, pyramidal cells in 7 of the 10 minicolumns in the memory pattern were assumed to require at least 10 spikes during the SOA trial, indicating substantial recurrent activity within the attractor memory.

The models were implemented using the CORTSIM library (manuscript forthcoming) that was written using the native Hoc and Mod languages of the parallel NEURON simulator, version 7.3 (Carnevale and Hines, 2006) and run on a Cray XC30 system. Construction of the model geometry, synaptic connection matrices and analysis of the spiking output from the NEURON simulation were done in Matlab. There were 25 trials in each trial set, which ran both with and without feedback connections, on both model 1 and model 2. Model 1 had a total of 39,424 neurons and each trial took about 4 min. to run on 256 cores. Model 2 had a total of 99,792 neurons and each trial took about 5 min. to run on 648 cores.

## Results

Both lateral inhibition in V1 and V2 and feedback from V2 were factors in the backward masking effects observed in the models. When the target and mask presentations were close in time and space, they mutually inhibited each other, first in V1 layer 4 and later in layer 2/3 and 5. As the SOA increased, the target pattern was more likely to become a fully activated attractor before the mask stimulus could begin to interfere via basket cells and di-synaptic inhibition. Feedback from V2 could reinforce the target attractor and be a factor in achieving full activation locally in V1 and regionally in both V1 and V2, if arrival was early enough, before the mask stimulus arrived to compete.

**Figure 4:**
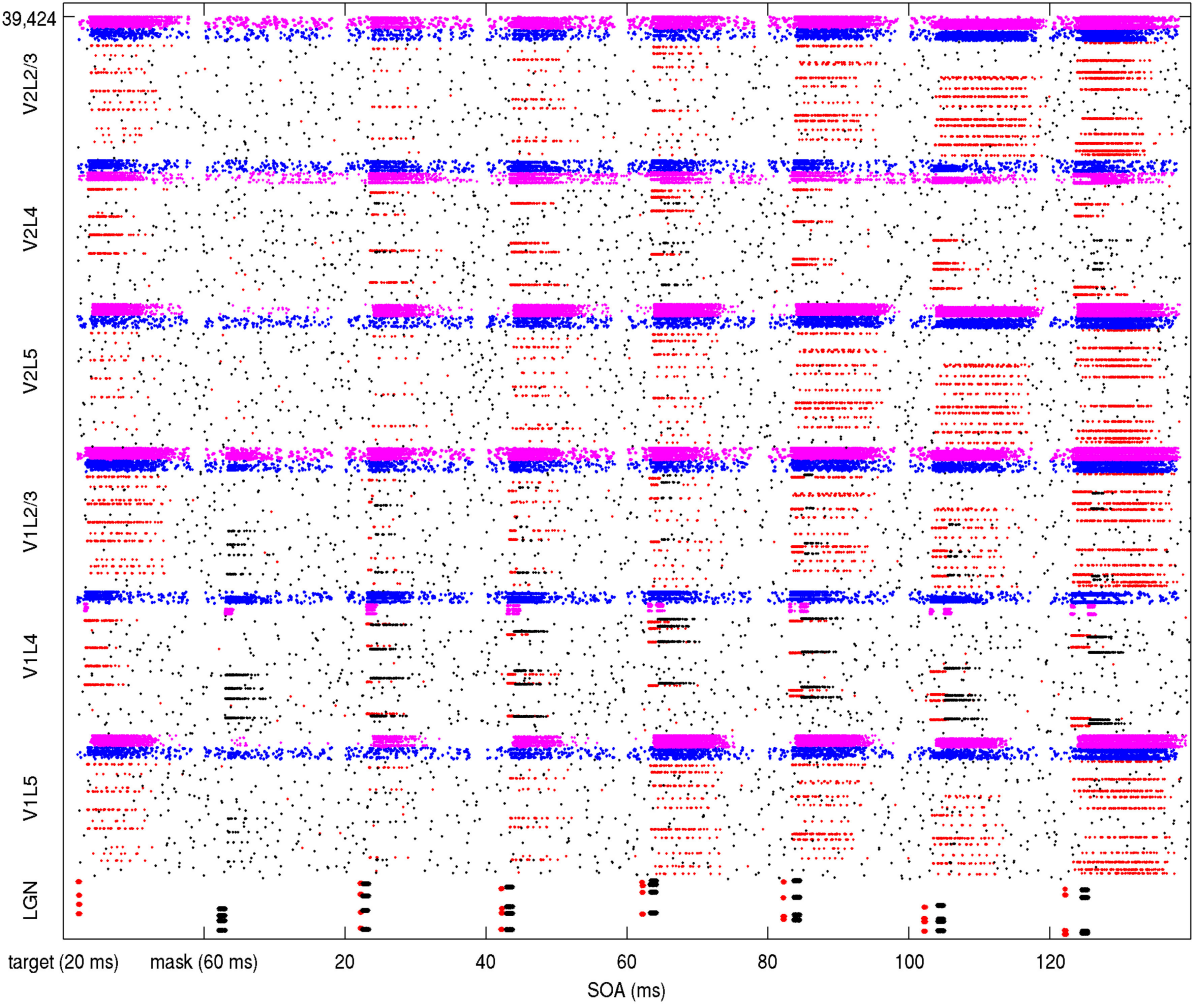
**Simulation of model 1 showing spiking activity during a backward masking trial with feedback projections in place**. The SOA was increased with a different target/mask presentation each second. The stimulated LGN target cells on the bottom are illustrated as red while the stimulated masking cells are black. In other areas, spiking pyramidal cells part of the target memory are illustrated as red while other pyramidals outside of the target memory are black. The pyramidal cells are within minicolumns, which can be seen when activated as red lines if in a target pattern and black lines if not. Each layer contains 256 minicolumns with 20 pyramidal cells, 32 basket cells, and 32 other interneurons. Spiking basket cells are shown in the figure as blue and spiking interneurons are magenta. Full activation of target patterns (where all 10 minicolumns can be seen as red lines) in both V1 and V2 can be seen in this trial at SOA intervals of 80, 100, and 120 ms.

**Figure 5:**
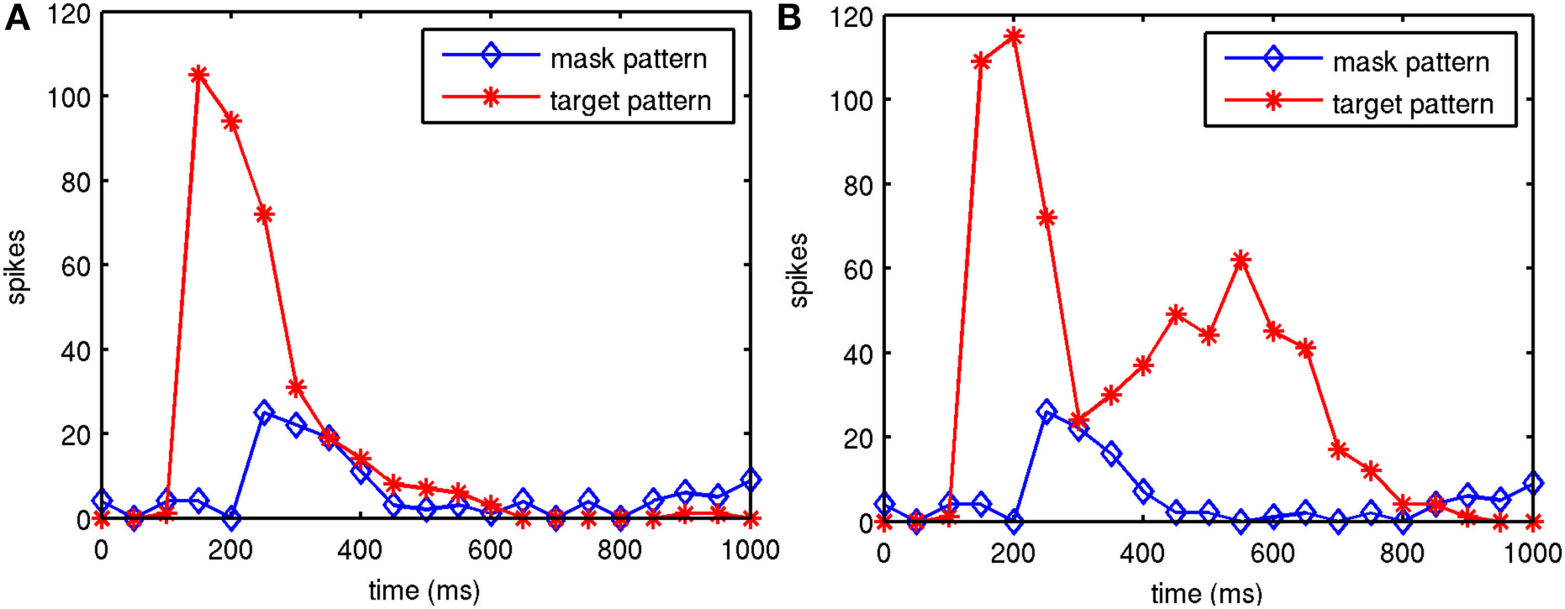
**Example of model 1 spiking activity in V1 layer 2/3 during two trials**. Shown are the target attractor and noise mask during backward masking trials, one with and one without feedback connections. Feedback activity reinforces and sustains the target attractor in the presence of the mask. Target stimulation starts at 100 ms, the SOA was 100 ms and the spikes were summed in 50 ms bins. **(A)** Without feedback from V2 to V1. **(B)** With feedback from V2 to V1.

The round-trip signaling latency of a target attractor in V1 feeding forward to V2 and feeding back to V1 is a minimum of about 25–40 ms, given a 10 ms latency of excitatory projections in each direction and synaptic integration at a single hop in V2. More robust feedback from V2 to V1 can take longer, once an attractor activates in V2. Other feedback can occur via secondary excitatory activity and pattern completion from other layers, but this can take longer, even more than 50 ms. The reason for this is not just the synaptic integration times of secondary, tertiary and greater hops, but the longer latencies of horizontal connections. From model 1 results, an example backward masking trial with feedback in place is shown in Figure 4. Results varied between trials from individual connection matrices and trial seed, but here full activation of the target pattern in layers 2/3 of V1 and V2 can be seen with an SOA of 80 ms and greater, with near activation at an SOA of 60 ms. This activation was due to competition in V1 layer 4 between the target and mask (red and black lines in area V1L4), allowing activity to propagate to V2. Following this, the recurrent feedback from V2 reinforced and sustained the activated target. Figure 5 shows this behavior as aggregated spiking activity on a different example, comparing trials with and without feedback connections.

Depending on the level of stimulus response and dynamics, some attractor memories did regionally complete in both V1 layer 2/3 and V2 layer 2/3 without feedback projections, but this activity was less likely than with feedback projections in place. Both excitatory and inhibitory feedback (via di-synaptic inhibition) from V2 contributed to enhancing the target attractor by increasing the likelihood of full activation of the target pattern.

**Figure 6:**
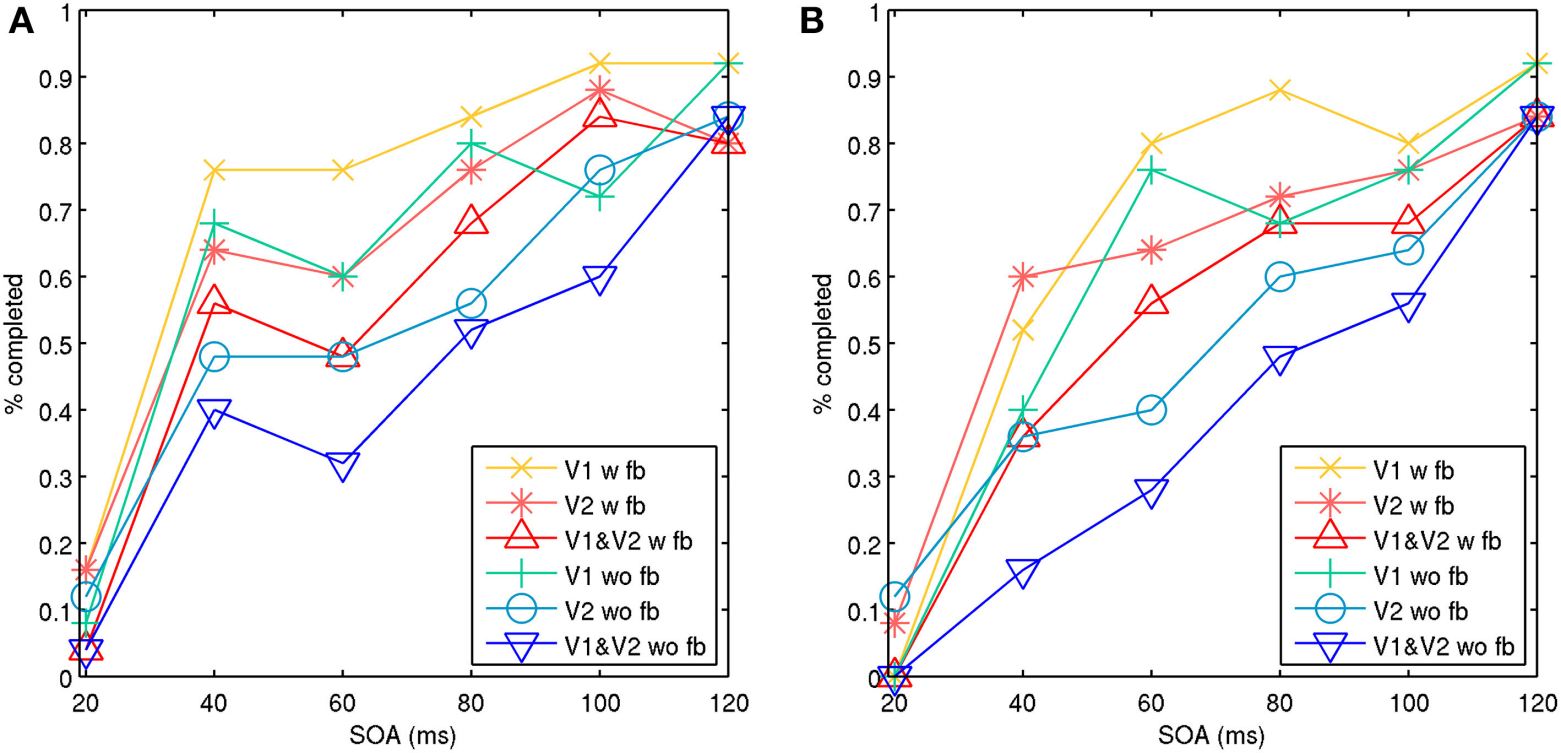
**Model 1 with noise masks showing aggregate percentage of targets completed in V1, V2, and both V1 and V2 with an increasing SOA**. The target and noise mask stimulation occurred in the same hypercolumn. Activity is shown with feedback connections (w fb) from V2 to V1 and without feedback connections (wo fb). The behavior represents type-A and type-B masking effects. Activation of both V1 and V2 represents activation across visual areas, which is assumed to be necessary for signal propagation up the visual stream to achieve conscious perception. **(A)** Illustrates aspects of a type-B masking with stimulation of four points for the target and noise mask. **(B)** Illustrates aspects of type-A masking with stimulation of four points for the target and five points for the noise mask, representing a higher masking salience.

Full target pattern activation usually took 25–50 ms or longer, depending on local connectivity and conduction strengths. Reinforcement of memory attractors from recurrent feedback sometimes needed to occur before a masking stimulus arrived, or the target attractor would be quiesced. Figure 6 shows aggregate results of two simulations for model 1, each consisting of 25 trials with feedback connections and 25 trials without. This demonstrated aspects of a type-B masking, as well as type-A masking at a higher noise salience, achieved by increasing the number of stimulated minicolumns in the noise mask from 4 to 5. With the presence of feedback connections, the masking effect was significantly reduced. The feedback connections also appeared to aid target pattern completion, and made the target attractor more stable. With the model 1 configuration and simulation assumptions, this shows that both lateral inhibition and recurrent feedback are factors in perception during metacontrast backward masking.

**Figure 7:**
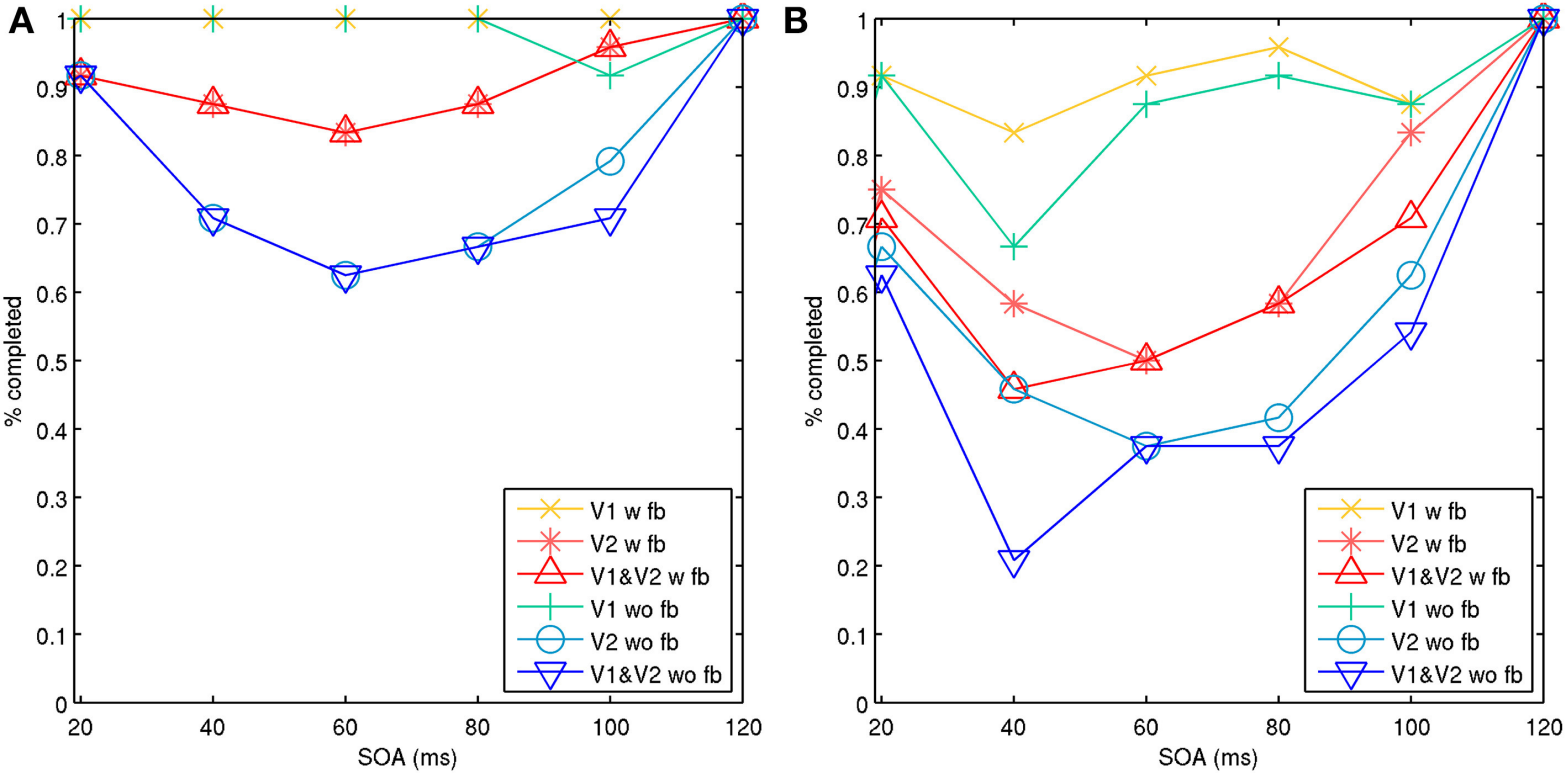
**Model 2 with spatial lines showing aggregate percentage of targets completed in V1, V2, and both V1 and V2 with increasing SOA**. Activity with feedback connections (w fb) from V2 to V1 and without feedback connections (wo fb). A vertical line target is masked with adjacent parallel lines on each side. **(A)** Target and mask lines were separated by three hypercolumns horizontally. **(B)** Target and mask lines were separated by one hypercolumn horizontally.

The model 2 configuration with the spatial line representations exhibited a type-B masking behavior or U-shaped function. Results can be seen in Figure 7, which shows simulations with the target and mask separated by a spatial distance of 1 and 3 hypercolumns. Results for each were aggregated across two sets of 25 trials, one with and one without feedback projections. The masking effects decreased with spatial distance, similar to psychophysical findings in Growney et al. (1977). Regional activity in layer 2/3 across both V1 and V2 produced type-B masking, as did analyzing activity in V2 alone. Feedback also diminished the masking effect on V2 alone, likely from boosted and recurrent feedforward activity from V1. However, when analyzing activity in V1 alone, activity appeared more monotonic when feedback is present.

## Discussion

The simulations showed that lateral, feedforward and feedback activity within V1 and V2 are all factors in activating and recognizing target patterns, in the presence of masks. Feedforward with feedback activity can also provide target reinforcement before lateral processing completes. This suggests that feedback processing reduces masking effects and correspondingly that masking effects may increase without the presence of feedback projections. This process of iterative reinforcement may occur among pairs of areas along the ventral stream. For example, V1 and V4 are also recurrently connected, and because of longer projection lengths, likely provides feedback with longer latencies. However, should higher level feature detectors be trained through experience or expectation to activate or reinforce an alternative lower representation, feedback interference could cause masking effects to increase on partial or ambiguous target stimuli. There is ongoing debate on the role of feedback processing on observed properties of backward masking (Di Lollo et al., 2002; Francis and Hermens, 2002; Põder et al., 2014), with object substitution in particular. The results here suggest there is a role, which might be more highlighted by contrasting expected sparse target recognition with ambiguous or conflicting (either primed or trained) higher level representations. On object substitution as defined by Di Lollo et al. (2000), feedback interference from larger set-sizes and distractors could be computationally explored with extensions to the existing model, by biasing or weakly stimulating higher-level attractor memories.

This study utilized a biophysical model, with equations for representing neural and synaptic properties, as well as microcircuits and network connectivity, from which characterized backward masking behaviors might emerge. Previous work has defined quantitative mathematical descriptions of backward masking behaviors from the top down. Quantitative mathematical methods known as efficient masking, mask blocking and target blocking have been described by Francis (2000) to account for type-B masking effects in metacontrast masking. Efficient masking refers to greater efficiency when masking at later SOAs when the target stimulus is weaker. The presented model did capture aspects of this behavior, because as the target attractor adapted through calcium dynamics and synaptic depression, lateral inhibition from the mask was more efficient at suppressing it. Mask blocking occurs if the target signal can block a weaker masking signal. This was observed as well, particularly at short SOAs. It may also have been a contributing factor to a sometimes observed target strength increase at an SOA of 40 ms, as seen in Figure 6A. A stimulated minicolumn in a target attractor memory is itself a small-world network and mini-attractor, which is more resilient to inhibition during stimulation and early activation. This resilience could be one explanation for sometimes observed higher target visibility during common-onset masking (Enns and Di Lollo, 2000), because the effective inhibition from the mask during target stimulation is lost, reducing the effective mask exposure length. Target blocking occurs when the mask is so strong, that the target signal cannot produce a percept. In the models, this can occur when lateral inhibition is high enough that not even the minicolumns can become recurrently active. Without active minicolumns, patch-level attractors cannot activate and complete.

Among computational models for backward masking, The BCS (Francis, 1997) and Bugmann and Taylor (2005) also used detailed neural representations. The BCS represents a complex hierarchy of feature detectors along the visual ventral stream with abstract non-spiking neurons, representing functional classifications of cells, including simple cells with two orientation preferences, as well as complex and hypercomplex cells. The BCS has been able to reproduce a broad range of psychophysical phenomena, including backward masking. It also has recurrent feedback and resonance with erosion, which may be a more abstract representation of distributed neural attractors and associated adaptation and dwell times. The spiking neural attractor model presented here is at a lower level of abstraction, representing various neural types, with functional activity and microcircuits determined by cell behavior and distributed synaptic connectivity. Functionally, it represents V1 and V2 and cannot yet reproduce the same level of behaviors as the BCS can. However, it likely has closer correspondence to spiking activity observed in electrophysiological studies of early visual cortex. It also has the potential of representing a large number of feature detectors for complex spatial representations, by scaling up the number of neurons and training the feature detectors as sparse, distributed neural codes. Bugmann and Taylor (2005) also developed a neural model for backward masking composed of a 5-level hierarchy of integrate-and-fire pyramidal cells. After initial stimulation of the LGN, each level extending across V1 and V2 received feedforward input. It did not have inhibitory neurons or feedback except for self-connections at the highest level, but was able to reproduce a U-shaped behavior response under some conditions, using this simplified model.

More biophysically detailed models can provide some unique advantages. They can allow for the exploration of some neural effects and relationships which cannot be easily investigated in electrophysiology experiments. The role of microcircuits in behavior can be investigated, as well as the effects of psychotropic drugs. For instance, the existence of synaptic channels in the models could enable the simulation of drug effects such as benzodiazepine on backward masking. Benzodiazepines have been found to slow down cortical processing and extend the attentional blink and other visual processing, both experimentally (Giersch and Herzog, 2004) and in computation models (Silverstein and Lansner, 2011). Thus, it could be predicted that benziodiazepines and other GABA agonists, which slow down cortical processing and feedback, would also increase the temporal window and SOA lengths when backward masking occurs. They may also amplify the depth of the masking function in type-B masking.

However, biophysically detailed neural models such as presented here have limitations and require a considerable amount of assumptions. These models can be very computationally intensive and may require parameter tuning. While some neural network parameters can be obtained from the literature, not all are well characterized, but the expectation is that biological plausibility constrains the hypotheses and parameter values enough that some evidence is gained on how the neural circuits might work. Some model assumptions were necessary, due to the limited electrophysiological data on primates and humans. In particular, the conductance strengths and ratios of excitatory and inhibitory feedforward and especially feedback projections are not well understood yet. This could be investigated further by computationally by varying the conductance strengths and excitatory/inhibitory ratios of these projections and observing changes in the masking function. Cell, synaptic and microcircuit parameters defined in the Appendix are based on experimental electrophysiology, but are simplified. In the models presented, not all neocortical layers and projections were represented in V1 and V2. Layer 6 was not implemented. Nor were there feedback connections between layer 2/3 and layer 5. In addition, because there were no areas represented downstream of V2, layer 5 of V2 did not have higher level feedback. To compensate for this, V2 layer 4 to layer 5 and V2 layer 4 to layer 2/3 conductance was boosted to provide a higher activity level. But regardless, the recurrent feedback did reduce the effects of backward masking, by making full target attractor activation more likely. A competing mask was also used with model 1, with slightly different results. At low SOAs, the target was usually quiesced as well, but at higher SOAs it was likely that both the target and competing mask would become active. However, target activity would be truncated after the competing metacontrast mask became active. This could be investigated further, as well as the effects of masks with partially overlapping features with the target. Such masks might have the effect of diminishing the masking effect, because the target attractor would receive more stimulation.

One weakness of the existing models is the limited spatial representation of feature detectors. Including biophysical feature detectors for various orientation preferences and contours is a challenging problem and an area for future work. Model 2 included spatial representations for lines as a step towards that goal. Extensions of the line representations may be applicable for computational investigations of the shine-through paradigm as discussed earlier, which is primarily based on the use of vertical lines. Model 1 often produced type-A masking, perhaps because the metacontrast noise mask was strong and in close spatial proximity. However, when the mask was weaker, it did sometimes produce aspects of type-B masking as well. Model 2 produced type-B masking under more parameter regimes, which may have occurred because the stimulated mask minicolumns were spatially farther away than in the model 1 configuration and therefore the lateral inhibition was weaker. When observing activity in V1 independently, type-A masking was more often produced. Yet, observing V2 alone more often demonstrated type-B masking behavior, as did co-activation of both V1 and V2. This may indicate type-B masking is a property of propagating attractor activity between V1 and V2. If so, stronger masks as used in model 1 may cause type-A masking overall because V1 is more strongly affected, causing highly diminished feedforward activity for propagation to V2. Weaker masks may allow more complex dynamics between V1 and V2, resulting in the emergence of type-B masking. Part of the U-shaped function may have occurred because of activity in layer 4, where the memory pattern long-range connections are weaker due to reduced lateral connectivity. This meant that activated minicolumns in layer 4 had shorter dwell times, and were more vulnerable sooner when the mask was presented.

It was also observed that lags in the inhibitory responses from the target and mask presentation during short SOAs can affect target salience. Adding a 3 ms delay on basket to pyramidal synapses made target pattern completion at short SOAs more likely. Lags in inhibitory populations can occur, because interneurons such as martinotti cells have facilitating synapses (Krishnamurthy et al., 2012) and gap junctions in basket cells can leak excitatory potentials to other basket cells. This might be a factor in common-onset masking (Enns and Di Lollo, 2000), since inhibitory populations can be largely silent before the common-onset stimulus.

### Conflict of interest statement

The author declares that the research was conducted in the absence of any commercial or financial relationships that could be construed as a potential conflict of interest.

## Appendix

### Cell models

The single cell models were described previously in Silverstein and Lansner (2011), where the implementation of the Hodgkin Huxley formalism (1952) was based on Ekeberg et al. (1991). With the membrane potential *V* and the Nernst potential *E_i_* for *i ϵ* { *Na, K, Ca, K_ca_}* and given Ohm's law: *I_i_ = g_i_(V−E_i_)* combined with Kirchoff's laws, yields:

$$\begin{array}{l} I_{m}=C_{m}\frac{dV}{dt}+g_{NA}\left(V,t\right)\left(V-E_{Na}\right)+g_{K}\left(V,t\right)\left(V-E_{K}\right)\\ \;+g_{Ca}\left(V,t\right)\left(V-E_{Ca}\right)+g_{K_{Ca}}\left(V,t\right)\left(V-E_{K_{Ca}}\right)+g_{L}\left(V-E_{L}\right) \end{array}$$

where *g_L_* is a constant leak conductance. The dynamic conductance *g_i_(V,t)* can be expressed with a gating model for individual ion channels. For modeling the for Na^+^ and K^+^ ion channel dynamics, Hodgkin and Huxley framework was employed.

$$I_{m}=C_{m}\frac{dV}{dt}+{\bar{g}}_{Na}m^{3}h\left(V-E_{Na}\right)+{\bar{g}}_{K}n^{4}\left(V-E_{K}\right)+g_{L}(V-E_{L})$$

where *g_i_* with *iϵ{Na, K}* is the maximal conductance when a channel is open, and gating variable *m* is Na^+^ channel activation, *n* is K^+^ channel activation *h* and is Na^+^ channel inactivation. The gating variables can be expressed as the following differential equations:

$$\begin{array}{l} \frac{dm}{dt}=\propto_{m}\left(1-m\right)-\beta_{m}m\;with\;\alpha_{m}\frac{A(V-B)}{1-e^{(B-V)/C}}\\ \;and\;\beta_{m}\frac{A(B-V)}{1-e^{(V-B)/C}}\\ \frac{dh}{dt}=\propto_{h}\left(1-h\right)-\beta_{h}h\;with\;\alpha_{h}\;\frac{A(B-V)}{1-e^{(V-B)/C}}\\ \;and\;\beta_{h}\frac{A}{1+e^{(B-V)/C}}\\ \frac{dn}{dt}=\propto_{n}\left(1-n\right)-\beta_{n}n\;with\;\alpha_{n}\;\frac{A(V-B)}{1-e^{(B-V)/C}}\\ \;and\;\beta_{n}\frac{A(B-V)}{1-e^{(V-B)/C}} \end{array}$$

A, B and C are parameters and independently specified for ∝ and β of each channel. Ca^2+^ is treated differently, because Ca^2+^ pools are assumed to be inside the cell near the cell membrane and can activate Ca^2+^ gated K^+^ channels to achieve hyperpolarization. Using *q* to represent Ca^2+^ activation, a relation similar to the Na^+^ channel activation (*m*) holds:

$$\begin{array}{l} \frac{dq}{dt}=\propto_{q}\left(1-q\right)-\beta_{q}q\;with\;\alpha_{q}\;\frac{A(V-B)}{1-e^{(B-V)/C}}\\ \;and\;\beta_{q}\frac{A(B-V)}{1-e^{(V-B)/C}} \end{array}$$

with the Ca^2+^ current into the cell being *I_Ca_ = g_Ca_q^5^V−E_Ca_)*. Channel equation parameters used in the simulations are specified in (Table A1).

**Table A1:** **Hodgkin-Huxley and NMDA ion channel parameters based on equations from Ekeberg et al. (1991) and values from Fransén and Lansner (1998)**.

		**Na^+ M^**	**Na^+ H^**	**K^+ N^**	**Ca^2^+ q**	**NMDA p**
α	A (mV^−1^ ms^−1^)	0.58	0.232	0.058	0.232	2.03 ms^−1^
	B (mV)	−50	−50	−50	10	–
	C (mV)	1	1	0.8	11	17
β	A (mV^−1^ ms^−1^)	0.174	1.16 (ms^−1^)	0.0145	0.0029	0.029 ms^−1^
	B (mV)	−59	−46	−40	10	–
	C (mV)	20	2	0.4	0.5	17

If we denote Ca^2+^ entering the cell as entering the *Ca_AP_* pool, then the change in concentration of [*Ca_AP_*] is equivalent to the rate of ions entering the pool and less the ions leaving the pool:

$$\frac{d\left[Ca_{AP}\right]}{dt}=\varphi_{AP}q^{5}\left(V-E_{Ca}\right)-\delta_{AP}\left[Ca_{AP}\right],$$

where ϕ_AP_ is the rate of Ca^2+^ influx and δ_AP_ is the rate of decay. The concentration [*Ca_AP_*] will activate Ca^2+^ gated K^+^ channels inside the cell membrane with the following current:

$$I_{K_{Ca}}={\bar{g}}_{K_{Ca}}\left(V-E_{K}\right)\left[Ca_{AP}\right]$$

After an increased neural firing rate, calcium buildup in the cell will cause hyperpolarization and a reduction in the firing rate. (Table A2) specifies neuron parameters and calcium dynamics. The [*Ca_AP_*] pool flux rates originate from either Ca^2+^ membrane channels (hh) or NMDA channels (NMDA).

**Table A2:** **Neuron parameters**.

**Parameter**	**Pyramidal**	**Basket**	**RSNP**	**Unit**
E_leak_	−64	−65	−65	*mV*
E_Na_	50	50	50	*mV*
E_Ca_	150	150	150	*mV*
E_K_	−80	−80	−80	*mV*
E_CA_(NMDA)	20	20	20	*mV*
C_m_	0.01	0.01	0.01	*μF/mm_2_*
g_m_	0.74	0.44	0.74	*μS/mm_2_*
g_na_ soma	150	150	150	*μS/mm_2_*
g_k_ soma	250 ± 2%	1000 ± 2%	1000 ± 2%	*μS/mm_2_*
g_na_ initial segment	2500	2500	2500	*μS/mm_2_*
g_k_ initial segment	83	5010	5010	*μS/mm_2_*
Ca_hh_ influx rate	1.00	1.00	1.00	*mV^−1^ms^−1^mm^−2^*
Ca_hh_ decay rate	6.3	9	30	*s*^−1^
Ca_NMDA_ influx rate	3.0	–	0.01	*s^−1^mV^−1^μS^−1^*
Ca_NMDA_ decay rate (soma)	1	–	3	*s*^−1^
Ca_NMDA_ decay rate (dend)	2	–	–	*s*^−1^
g_k_ (Ca)	2.9	0.15	0.29	*pS / mM*
soma diameter ± stdev	21 ± 2.1	7 ± 0.7	7 ± 0.7	*μm*
Total compartments	4	3	3	
Dendritic area (rel. soma)	4	4	4	
Initial seg area (rel. soma)	0.1	0.1	0.1	

### Synaptic equations

For implementing the synaptic coupling, neurotransmitter gated ionotropic synapses were modeled, where the channels conduct ionic current produced by a voltage driving force and channel conductance. AMPA and GABA_A_ currents are governed by:

$$I_{syn}=\left(E_{syn}-V\right)G_{syn}s\;0\leq s\leq 1$$

Where *s* is the level of synaptic activation, with 1 being the most active. All synapses are consolidating and saturating as defined by Lytton (1996) and depressing as defined by Varela et al. (1997). Every synaptic spike results in neurotransmitter release for duration *C_dur_* when it binds to receptors with binding rate ∝ and unbinding rate β. Saturation occurs because any spike following another spike by less than *C_dur_* extends neurotransmitter release for another *C_dur_* interval. *W_sum_* is the sum of all synaptic weights currently active within *C_dur_*. After each spike and during *C_dur_, W_sum_* is incremented by the synaptic weight *W_syn_* and after *C_dur_, W_sum_* is decremented by *W_syn_*. Consolidation occurs by summing across synaptic activations into state variables *R_on_* and *R_off_*, which have the following dynamics:

$$\frac{dR_{on}}{dt}=\frac{W_{sum}R_{inf}-R_{on}}{R_{tau}}\;\frac{dR_{off}}{dt}=-\beta R_{off}\;R_{inf}=\frac{\propto }{\propto +\beta }$$

The consolidated level of synaptic activation is represented by *s =R_on_ + R_off_*. For synaptic depression, *W_syn_* is decreased during *C_dur_* according to recent short-term pre-synaptic activity with: *W_syn_ = W_syn_d_fast_d_slow_*, where depression variable *d_i_ = d_i_D_i_* after a spike occurs, which then decays to 1 with *d_i_* = 1−(1−*d_i_)e^−t/τ^i*. NMDA synapses are similar to AMPA and GABA_A_ but with additional dynamics for the Mg^2+^ block. Parameters for synaptic dynamics are specified in (Table A3).

$$I_{NMDA}=\left(E_{NMDA}-V\right)G_{NMDA}ps\;0\leq s\leq 1\;0\leq p\leq 1$$

**Table A3:** **Parameters for synaptic dynamics**.

**Pre-Post**	**Type**	***C_dur_sec***	***τ_nraise_sec***	***τ_decay_sec***	***E_rev_mV***	***D_fast_***	***D_slow_***	***τ_rec_ ms***	***τ_rec_ ms***	***E_slow_ mV***
Pyr-Pyr	Kainate/AMPA	0.0	0.0	0.006	0	0.78	0.98	634	9200	–
Pyr-Pyr	NMDA	0.02	0.005	0.150	0	0.78	–	634	–	0.020
Pyr-Basket	Kainate/AMPA	0.0	0.0	0.006	0	0.78	0.98	634	9200	–
Basket-Pyr	GABA_A_	0.0	0.0	0.006	−85	0.94	–	1900	–	–
Pyr-Rsnp	Kainate/AMPA	0.0	0.0	0.006	0	0.75	0.98	575	9200	
Pyr-Rsnp	NMDA	0.02	0.005	0.150	0	0.75	–	575	–	0.020
Noise	Kainate/AMPA	0.0	0.0	0.01	0	–	–	–	–	–

Where *p* is the voltage gated variable for the Mg^2+^ block with the following dynamics:

$$\frac{dp}{dt}=\propto_{p}\left(1-p\right)-\beta_{p}p\;with\;\alpha_{p}=A_{\propto }e^{\frac{V}{C}}\;\beta_{p}=Ae^{-V/C}$$

The parameters A and C are independently specified in (Table A1) for ∝ and β of channel *p*. All neurons but the LGN relay cells receive noise input from an excitatory synapse driven by a 300 Hz Poisson process. The pyramidal cell has the noise synapse on the apical dendrite and the basket and RSNP cells have it on the basal dendrite. The noise synapse is identical to the AMPA synapse but without synaptic depression, and a decay time constant of 10 ms.

### Network model

The network architecture is organized into interconnected patches for LGN, V1 and V2, with some differences between models 1 and 2. The fixed neuron counts for patches V1 and V2 can be found in (Table A4). Within patches V1 and V2, individual minicolumns span across layers 2/3, 4, and 5. Local populations of pyramidal cells in each of these layers are interconnected with local populations in the other layers, with the pre-synaptic to post-synaptic connection probabilities listed in (Table A5). These probabilities were partially determined by tuning for plausible attractor activity levels in individual layers when stimulated with targets.

**Table A4:** **V1 and V2 neuron counts**.

**layer**	**Neuron**	**No. in minicolumn**	**No. in model 1 hyercolumn**	**No. in model 2 hypercolumn**	**Model 1 total**	**Model 2 total**
L 2/3	pyramidal	20	320	160	5120	12960
L 2/3	basket	–	32	16	512	1296
L 2/3	rsnp	2	32	16	512	1296
L 4	pyramidal	20	320	160	5120	12960
L 4	lg. basket	–	32	16	512	1296
L 4	sm. basket	2	32	16	512	1296
L 5	pyramidal	20	320	160	5120	12960
L 5	basket	–	32	16	512	1296
L5	rsnp	2	32	16	512	1296

**Table A5:** **Synaptic connection probabilities between pyramidal cells in the different layers within the minicolumns**.

**src. layer**	**dst. layer**	**conn. prob. model 1 (%)**	**conn. prob. model 2 (%)**
V1L4	V1L2/3	13	8
V1L4	V1L5	26	16
V1L5	V1L2/3	6	3
V2L4	V2L2/3	13	12
V2L4	V2L5	34	21
V2L5	V2L23	6	3

Between patches V1 and V2 are feedforward and feedback memory pattern projections, which can be excitatory or inhibitory. Excitatory projections connect a subset of minicolumns within two individual attractor memories across two regions. Inhibitory projections connect one attractor memory to other attractor memories in common hypercolumns that also receive an excitatory projection from the originating attractor memory, potentially inhibiting these other attractor memories through di-synaptic inhibition. The expected synaptic counts in projections between pairs of attractor memories and opposing attractor memories (within common hypercolumns) are listed in (Table A6). The excitatory synaptic counts were tuned to provide plausible activity transfer from stimulated attractor memories. The inhibitory synaptic counts were generally assumed to be about 20% of excitatory synaptic counts for feedforward projections and about 40–60% of excitatory synaptic counts for feedback projections.

**Table A6:** **Expected approximate feedforward (ff) and feedback (fb) projection synapse counts between attractor memories**.

**proj. src**.	**proj. dst**.	**dir**.	**exc. syn model 1**.	**inh. syn model 1**.	**exc. syn model 2**.	**inh. syn model 2**.
V1L4	V2L4	ff	560	112	800	160
V1L2/3	V2L2/3	ff	100	20	100	20
V2L2/3	V1L2/3	fb	32	16	32	14
V2L4	V1L2/3	fb	32	16	32	14
V2L4	V1L5	fb	256	128	256	102
V2L4	V2L5	fb	512	256	512	205

*Includes both excitatory (exc) synapses on pyramidal cells and inhibitory (inh) synapses on RSNP interneurons. The V2L4 to V2L5 projection partially compensates for the lack of feedback to V2 from higher areas*.
